# Supplementary material for: Critical role of CD4+ T cells and IFNγ signaling in antibody-mediated resistance to Zika virus infection
Source: Nat Commun. 2018 Aug 7;9:3136. doi: 10.1038/s41467-018-05519-4 (PMC6081430; doi:10.1038/s41467-018-05519-4)
Supplement: Supplementary file 1 — Supplementary Information [file 41467_2018_5519_MOESM1_ESM.pdf]

Supplementary Information for ‘‘Critical role of CD4$^+$ T cells and IFNγ signaling in antibody-mediated resistance to Zika-Virus infection’’ by Lucas, kitoko et al.

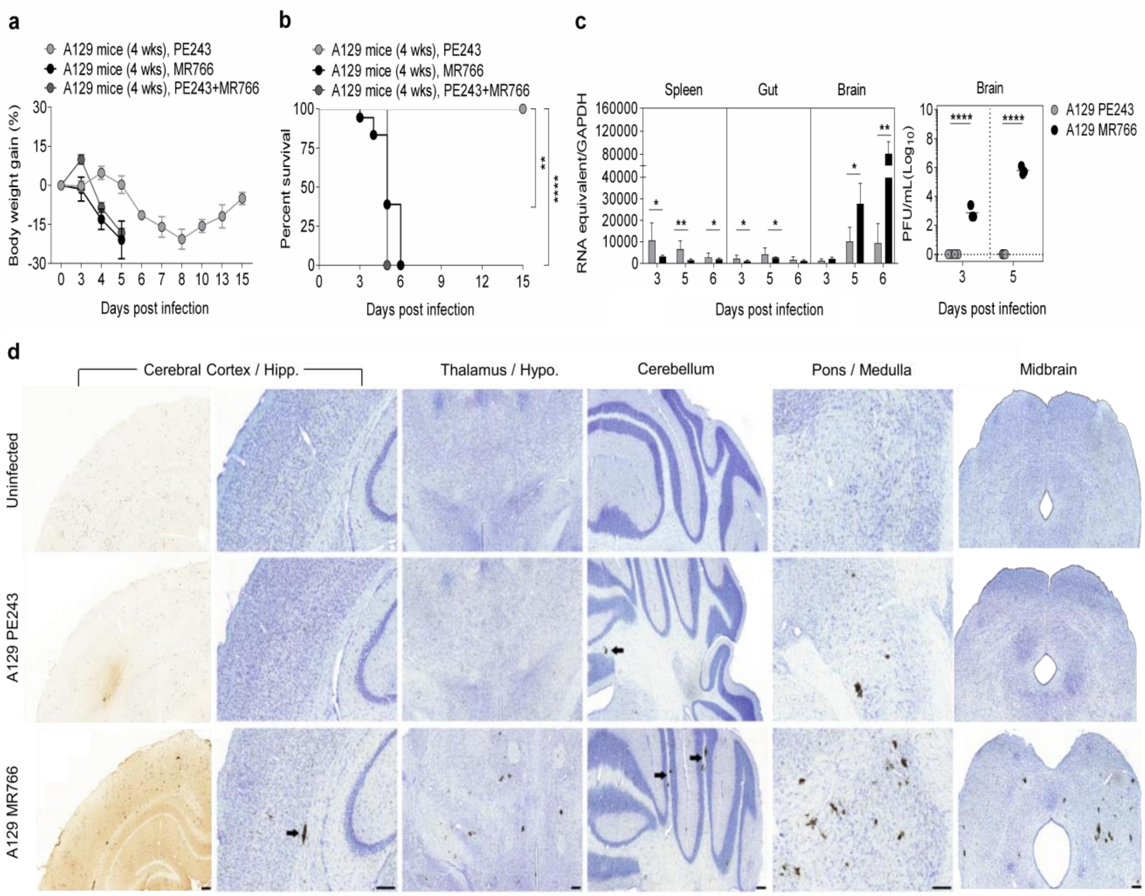

Supplementary Fig. 1 ZIKV MR766 induces a lethal infection in A129 mice. Four week old A129 mice were inoculated intravenously with 2 $\times 10^5$ PFU of ZIKV PE243 (n = 7), MR766 (n = 26), or both (n = 4). All infected mice were monitored for up to 15 days post infection. **a** body weight gain and **b** lethality of A129 infected mice. **c** ZIKV RNA copies in the spleen, gut, and brain at days 3, 5 and 6 post infection and presence of infectious particles of ZIKV in the brain at 3 and 5 days post infection. The horizontal dotted line indicates the limit of detection. **d** Representative photomicrographs showing the pattern of IgG staining (in brown) and DAB staining in coronal brain sections obtained from ZIKV PE243 or MR766-infected mice at day 5 post infection. Uninfected mice were used as control. The arrow points to a focus of microhemorrhage in the white matter. Scale bar: 200 μm. Results are shown only as mean in **c** or as mean ± standard deviation in **a** and **c**. Data are presented as a pool of two to four independent experiments in **a** and **b**, experiment were performed once in **c**. Survival data were analyzed by Log rank test. Data were analyzed by Student’s t-test. (*) p≤ .05; (**) p ≤ .01; (****) p ≤ .0001. Hypo, hypothalamus; hipp., hippocampus.

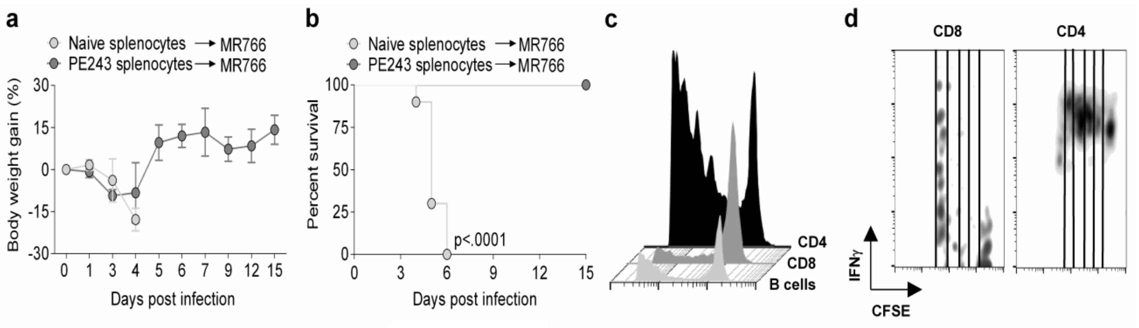

Supplementary Fig. 2 ZIKV PE243-immune splenocytes protect against ZIKV MR766. Recipient A129 mice were inoculated intravenously with 2×10^5 PFU of lethal ZIKV MR766 simultaneously to the receipt of splenocytes obtained from ZIKV strain PE243 infected-A129 donor mice at 7 days post infection. Uninfected mice were used as donor of naive splenocytes. **a** Body weight gain and **b** lethality of A129 recipient mice that received PE243-immune (n = 9) or naive splenocytes (5×10^7/mouse) (n = 10) monitored for up to 15 days post infection. **c** Donor PE243-immune splenocytes were stained with CFSE to assess proliferation of donor B cells, $CD4^+$ and $CD8^+$ T cells and **d** IFNγ producing $CD4^+$ and $CD8^+$ T cells in the spleen of recipient mice by flow cytometry at 3 days post infection. Survival data were analyzed by Log rank test. Results are shown as mean ± standard deviation in **a**. Data are representative of four independent experiments in **a** and **b** or two independent experiments in **c** and **d**.

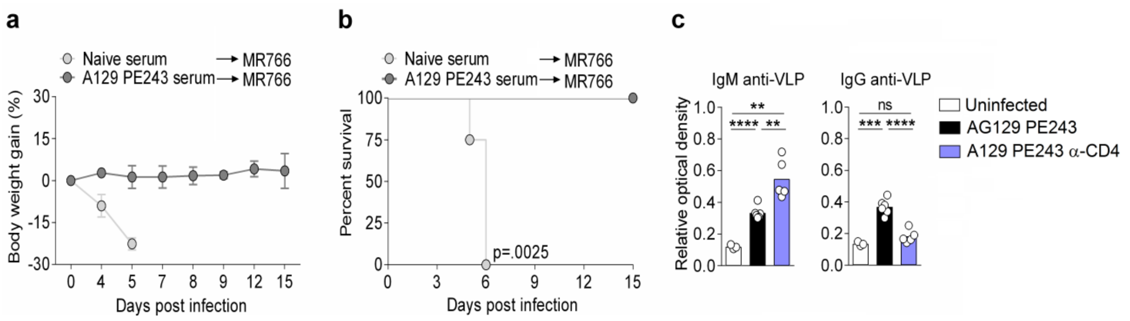

Supplementary Fig. 3 ZIKV PE243-immune serum protects against ZIKV MR766. Serum obtained from ZIKV PE243-infected A129 mice at 7 days post infection were administrated intravenously (150–200 μL) to ZIKV MR766-infected A129 mice (2×10^5 PFU, i.v). Uninfected A129 mice were used as donor of naive serum. **a** Body weight gain and **b** lethality of recipient mice that received A129 PE243 serum (n = 8) or naive serum (n = 4) were monitored for up to 15 days post infection. **c** Serum IgM and IgG anti-ZIKV VLPs in uninfected mice (n = 3), ZIKV PE243-infected AG129 mice (n = 6) and ZIKV PE243-infected A129 mice previously depleted of $CD4^+$ T cells (n = 5). Results are represented as relative optical density by performing the ratio with ZIKV strain PE243-infected A129 mean values for IgG or IgM ($\frac{x\ group\ O.D.sum\ value}{A129\ PE243\ O.D.sum\ mean\ value}$). Results are shown only as mean in **c** or as mean ± standard deviation in **a**. Data are representative of four independent experiments in **a** and **b** or two independent experiments in **c**. Survival data were analyzed by Log rank test. Data were analyzed by Student's t-test in **c**. (**) p≤ .01.; (***) p ≤ .001; (****) p ≤ .0001; not significant (ns).

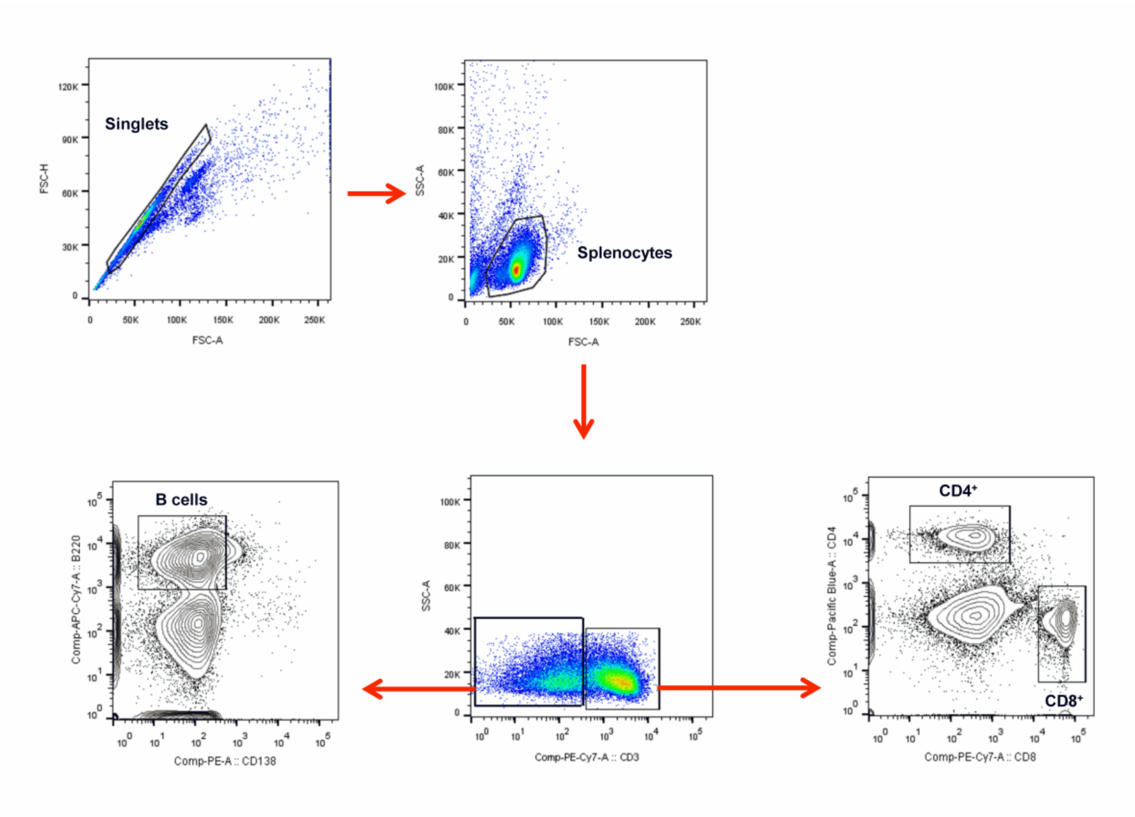

Supplementary Fig. 4 Gate strategies for lymphocyte isolation by FACS. Single cells were gated based on FSC-H x FSC-A plot. Inside single cells, splenocytes were gated in a SSC-A x FSC-A plot. $CD3^{+}$ and $CD3^{-}$ populations were gated inside splenocytes. $CD4^{+}CD8^{-}$ ($CD4^{+}$ T cells) and $CD8^{+}CD4^{-}$ ($CD8^{+}$ T cells) populations were obtained inside $CD3^{+}$ gate, while $B220^{+}CD138^{-}$ (B cells) population was obtained inside $CD3^{-}$ gate.

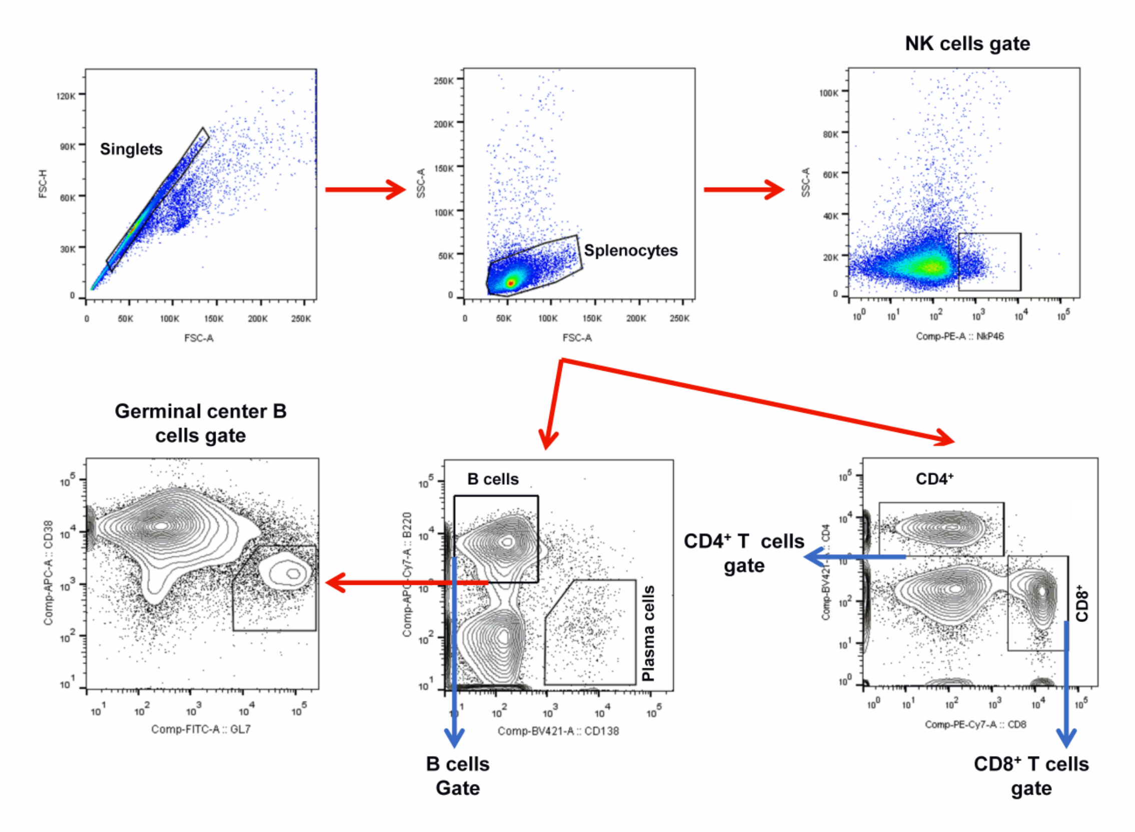

Supplementary Fig. 5 Gate strategies for lymphocytes analyses. Single cells were gated based on FSC-H x FSC-A plot. Inside single cells, splenocytes were gated in a SSC-A x FSC-A plot. Five distinct populations were analyzed inside splenocytes, NKp46$^+$ (NK cells), CD4$^+$CD8$^-$ (CD4$^+$ T cells), CD8$^+$CD4$^-$ (CD8$^+$ T cells), B220$^+$CD138$^-$ (B cells), and B220$^-$CD138$^+$ (plasma cells). Germinal center B cells were analyzed inside B cells gate. Whenever indicate in the results section, cytokines, cytotoxic factors, transcription factors, activation markers or subpopulations of lymphocytes were analyzed inside the populations showed above.

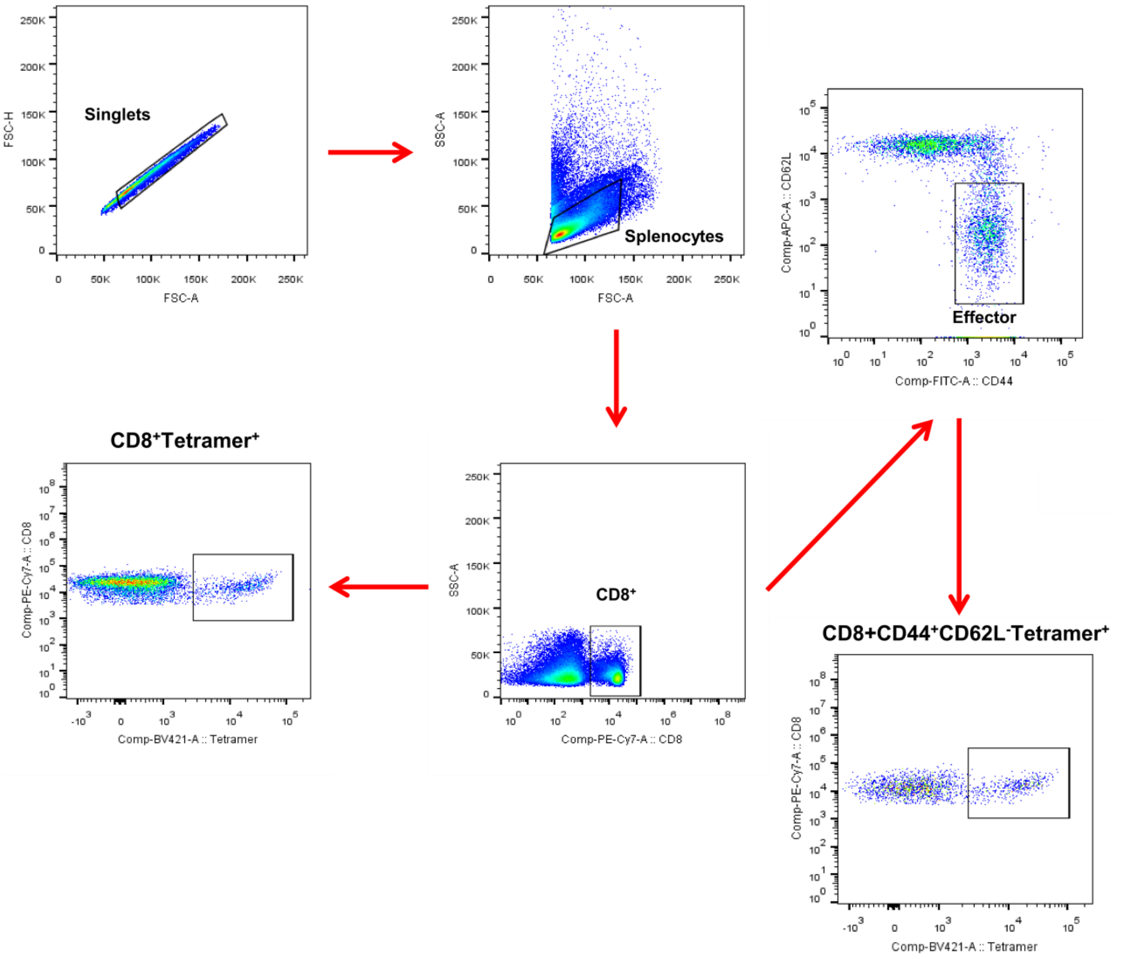

Supplementary Fig. 6 Gate strategies for protein E tetramer analyses. Single cells were gated based on FSC-H x FSC-A plot. Inside single cells, splenocytes were gated in a SSC-A x FSC-A plot. $CD8^+$ T cells population ($CD8^+$) was analyzed inside splenocytes gate. Tetramer positive cells were analyzed inside total $CD8^+$ or effector $CD8^+CD44^+CD62L^-$ T cell populations.

ANTIBODIES	DILUTION	SOURCE	REF. NUMBER
Anti-B220-BV605	1:200	(BioLegend Clone: RA3-6B2)	Cat# 103244
Anti-CD107a-Alexa Fluor 488	1:100	(eBioscience Clone: eBio1D4B)	Cat# 53-1071-82
Anti-CD138-BV421	1:200	(BioLegend Clone: 281-2)	Cat# 142523
Anti-CD185 (CXCR5)-PE	1:200	(BioLegend Clone: L138D7)	Cat# 145503
Anti-CD25-APC	1:200	(eBioscience Clone: PC61.5)	Cat# 17-0251-81A
Anti-CD3-PEcy7	1:200	(eBioscience Clone: 145-2C11)	Cat# 25-0031-82
Anti-CD38-APC	1:200	(BioLegend Clone: 90)	Cat# 102712
Anti-CD4-eFluor450	1:200	(eBioscience Clone: RM4-5)	Cat# 48-0042-82
Anti-CD4-APC	1:200	(eBioscience Clone: GK1.5)	Cat# 17-0041-82
Anti-CD4-PEcy5	1:200	(eBioscience Clone: GK1.5)	Cat# 15-0041-82
Anti-CD4-PE	1:200	(eBioscience Clone: RM4-5)	Cat# 12-0043-82
Anti-CD44-PerCP-Cy5.5	1:200	(BioLegend Clone: IM7)	Cat# 103032
Anti-CD44-PE	1:200	(eBioscience Clone: IM7)	Cat# 12-0441-82
Anti-CD62L-BV421	1:200	(BioLegend Clone: MEL-14)	Cat# 104436
Anti-CD62L-APC	1:200	(Tonbo biosciences Clone: MEL-14)	Cat# 20-0621-U100
Anti-CD8α-PEcy7	1:200	(Tonbo biosciences Clone: 53-6.7)	Cat# 60-0081-U100
Anti-CD8α-APC	1:200	(eBioscience Clone: 53-6.7)	Cat# 17-0081-81
Anti-GL-7-Alexa Fluor 488	1:200	(BioLegend Clone: GL7)	Cat# 144612
Anti-IgG Fc-PE	1:200	(Jackson ImmunoResearch Lab)	-------------------------
Anti-NKp46-PE	1:80	(eBioscience Clone: 29A14)	Cat# 12-3351-82
Anti-PD-1-BV605	1:80	(BioLegend Clone: 29F.1A12)	Cat# 135219

Supplementary Table 1 Antibodies used for cell surface staining. Monoclonal antibodies conjugated with fluorochromes used for lymphocyte surface's immunophenotyping and dilution, source, and reference numbers of antibodies.

ANTIBODIES	DILUTION	SOURCE	REF. NUMBER
Anti-Foxp3-Alexa Fluor 488	1:100	(Thermo Fisher Scientific Clone: FJK-16s)	Cat# 53-5773-80
Anti-Grzm B-Alexa Fluor 647	1:200	(BioLegend Clone: GB11)	Cat# 515405
Anti-IFNγ-APC	1:100	(Tonbo biosciences Clone: XMG1.2)	Cat# 20-7311-U100
Anti-IL-17-PE	1:100	(eBioscience Clone: eBio 17B7)	Cat# 12-7177-81
Anti-IL-2-PE	1:100	(BD Pharmingen Clone: JES6-5H4)	Cat# 18175Z
Anti-IL-4-APC	1:100	(Tonbo biosciences Clone: 11B11	Cat# 20-7041-U100
Anti-Perforin-PE	1:100	(eBioscience Clone: eBioMAK-D)	Cat# 12-9392-80
Anti-TNFα-PE	1:100	(eBioscience Clone: MP6-XT22)	Cat# 12-7321-82

Supplementary Table 2 Antibodies used for staining of permeabilized cells. Monoclonal antibodies conjugated with fluorochromes used for lymphocyte internal immunophenotyping and dilution, source, and reference numbers of antibodies.
